# Ultra-low dose of rituximab in rheumatoid arthritis: study protocol for a randomised controlled trial

**DOI:** 10.1186/s13063-017-2134-x

**Published:** 2017-08-30

**Authors:** Alfons A. den Broeder, Lise M. Verhoef, Jaap Fransen, Rogier Thurlings, Bart J. F. van den Bemt, Steven Teerenstra, Nadine Boers, Nathan den Broeder, Frank H. J. van den Hoogen

**Affiliations:** 1 Department of Rheumatology, Sint Maartenskliniek, PO Box 9011, 6500 GM Nijmegen, The Netherlands; 2 Department of Rheumatology, Radboudumc, Nijmegen, The Netherlands; 3 Department of Pharmacy, Sint Maartenskliniek, Nijmegen, The Netherlands; 4 Department of Pharmacy, Radboudumc, Nijmegen, The Netherlands; 5 Department of for Health Evidence, Section of biostatistics, Radboudumc, Nijmegen, The Netherlands

**Keywords:** Rheumatoid arthritis, Dose reduction, Rituximab, Low dose, Retreatment, Randomised controlled trial, Non-inferiority, Design, Decremental cost-effectiveness ratio (DCER)

## Abstract

**Background:**

A standard low-dosing schedule of rituximab (RTX; 2 × 500 mg or 1 × 1000 mg) is as effective for active rheumatoid arthritis (RA) as the registered dose (2 × 1000 mg). Moreover, several small uncontrolled studies suggest that even lower-dosed treatment with RTX also leads to good treatment response in patients with RA. Retreatment with such an ‘ultra-low’ dose RTX in patients who responded well to RTX induction treatment is of special interest, as long-term use of lower RTX doses may lead to shorter infusion duration, lower risk of adverse events and lower costs. However, the effect of ultra-low dose of RTX has not been investigated using a controlled trial of proper design and dimensions.

**Methods/Design:**

REDO is an investigator driven six-month pragmatic, double-blind, randomised controlled non-inferiority trial on the effects of ultra-low-dose RTX (1 × 500 or 1 × 200 mg) compared to standard low dose (1 × 1000 mg) in RA patients who are being retreated with RTX. A total of 140 RA patients, having reached low disease activity (DAS28CRP < 2.9) after the previous RTX infusion and DAS28CRP < 3.5 at moment of retreatment, are randomised in a ratio of 1:2:2 to 1 × 1000 mg, 1 × 500 mg or 1 × 200 mg. The primary objective is testing non-inferiority of the ultra-low-dose vs. standard low-dose RTX, by comparing mean change in DAS28CRP from baseline to six months to the non-inferiority margin of 0.6. Secondary outcomes over the same period are: function; quality of life; safety; costs; and pharmacokinetics and dynamics as process measures.

**Discussion:**

This study protocol shares characteristics of both early dose finding trials as well as late pragmatic clinical studies. Several choices in the design of this trial are described and possible consequences for RA treatment and expected biosimilar introduction are discussed.

**Trial registration:**

Dutch Trial Register, NTR6117. Registered on 15 November 2016 (CMO NL57520.091.16, 8 November 2016)

**Electronic supplementary material:**

The online version of this article (doi:10.1186/s13063-017-2134-x) contains supplementary material, which is available to authorized users.

## Background

Rituximab (RTX) is a chimeric anti-CD20 monoclonal antibody authorised for use in patients with severe active rheumatoid arthritis (RA) in combination with methotrexate (MTX) when patients have an inadequate response or intolerance to other disease-modifying antirheumatic drugs (DMARDs), including one or more tumour necrosis factor inhibitors (TNFi). Two large systematic reviews confirmed the effectiveness of RTX in patients with RA in combination with MTX compared to MTX alone [1, 2]. In addition, long-term safety has been confirmed up to 11 years, with infection risk comparable to other biological DMARDS (bDMARDs) [3, 4].

The dose-finding phase of RTX has some interesting aspects. Since RTX was originally developed as a treatment for non-Hodgkin’s lymphoma, its optimal dose was initially determined for that indication [5]. The first two studies of RTX in RA indeed used treatment protocols based on experience in the treatment of lymphoma [6, 7]. Both studies were open-label and consisted of a limited number of patients. It was reasoned that RA could be seen as a low-grade lymphoma of synovial tissue, caused by an oligoclonal (instead of monoclonal) proliferation of B cells exhibiting malignant behaviour by destroying local tissues. Using this comparison, patients were treated with a single remission-induction treatment course, identical to that for non-Hodgkin’s lymphoma, combining four weekly RTX infusions of 750 mg/m^2^ with prednisone and cyclophosphamide. The treatment goal was to achieve disease remission by eradication of pathogenic B cells. Only adriamycin was omitted as co-medication to decrease the chance on treatment-related side toxicity. These two open-label case series showed that a single RTX-based treatment course could induce disease remission in a proportion of patients with RA. Although no formal dose-finding efforts were done, Leandro et al. concluded in their uncontrolled study of 22 RA patients that doses below 600 mg/m^2^ were less effective, but this conclusion was based on only four patients. The first randomised controlled trial (RCT) to examine the efficacy of RTX in RA patients aimed at obtaining a treatment regimen without cyclophosphamide instead of dose-finding and used a simplified RTX dosing regimen of 1000 mg on treatment days 1 and 15 [8]. This dose is now the registered dose for treatment of RA patients.

Thereafter, dosing schedules of 2 × 500 mg and 1 × 1000 mg have been tested in several phase-three and phase-four studies; a recent large systematic review showed that these were non-inferior to regular-dose RTX. Therefore, the current recommended RTX doses are 2 × 500 mg or 1 × 1000 mg (standard low-dose RTX) at least every six months. The second infusion is commonly given with an interval of two weeks (e.g. for 2 × 1000 mg) [9]. Although there have been no high-quality strategy studies to establish what is the best retreatment strategy, either fixed six-month interval retreatment or disease activity guided treat-to-target retreatment seem the optimal strategy.

However, even lower doses of RTX may be effective for treatment of RA. In three case studies, ultra-low doses of RTX (1 × 50 to 2 × 100 mg) were surprisingly associated with deep peripheral B-cell depletion and, in general, adequate RA disease control [10–12]. Adding to these observations, a recent small, prospective open label study in 14 RA patients showed that a single dose of 100 mg RTX led to peripheral B-cell depletion in 11 patients (79%) after two weeks [13]. In that study, mean (± SEM) DAS28 score of all patients decreased from 6.2 ± 0.8 at baseline to 2.9 ± 0.8 at 24 weeks after infusion, although two patients needed additional RTX treatment.

The use of ultra-low-dose RTX for retreatment could especially be effective. First, B-cell depletion by RTX can persist during the entire interval between infusions [8, 14]. It was shown that that lower baseline B-cell counts were associated with complete B-cell depletion following a first 500-mg dose of RTX [15]. This suggests that the (partially) persisting B-cell depletion induced by an earlier infusion could reduce the dose of RTX needed for retreatment infusions.

A final argument for possible effectiveness of ultra-low-dose RTX is the fact that similar monoclonal antibodies have been shown to be effective well below the authorised doses for RTX. For ocrelizumab and ofatumumab, two humanized anti-CD20 monoclonal antibodies, it was concluded that doses of 2 × 200 mg and 2 × 300 mg, respectively, provide optimal B-cell depletion as well as the best clinical responses [16]. Although these much lower doses compared to RTX might also be possible due to higher affinity or cytotoxic efficacy of the drug, it lends further credibility to study the efficacy of similar ‘ultra-low’ doses of RTX.

The use of ultra-low RTX could present several advantages over standard low-dose RTX. First, infection risk should be lower, as RTX use is associated with a dose-dependent – although still low – risk of serious infection [17, 18]. Also, shorter infusion duration and less administered drug could lead to less patient burden and perhaps lower risk for infusion reaction [19]. Further, RTX treatment currently is relatively expensive, with costs for low-dose 1 × 1000 mg every six months being in the range of €4000–7000 per year. Although RTX was proven to be cost-effective in patients with an inadequate response to TNFi [20], use of ultra-low doses will further decrease costs and thereby improve cost-effectiveness. A combination of a possible effective dose of 200 mg every six months and expected price reductions due to upcoming availability of a rituximab biosimilar, could result in a bDMARD option availability for under €1000 per patient per year.

The use of an ultra-low dose of RTX might, however, also lead to increased disease activity in the subset of patients whose minimal effective RTX dose is 1000 mg. Therefore, prediction of response to ultra-low-dose RTX would be key to prevent patients from flaring experiencing accelerated joint damage [21]. Interesting baseline (at the moment of considering RTX retreatment) candidates for predicting the chance of good response on an ultra-low dose include higher RTX drug levels, absence of anti-RTX-antibody levels and low peripheral B-cell counts, as it might be hypothesised that these are all indicators for lower RTX need [22].

In conclusion, although the use of ultra-low doses of RTX seems promising, its effects have never been studied in a trial of proper design and size. We therefore aim to perform a RCT to study whether retreatment with one of two ultra-low RTX doses (1 × 200 mg or 1 × 500 mg) is non-inferior to retreatment with the standard low-dose RTX (1 × 1000 mg) for patients with RA who were already successfully treated with standard low-dose RTX. Also, we will analyse whether there are differences between retreatment with ultra-low dose and standard low dose in the occurrence of serious and non-serious adverse events and cost-effectiveness, and we will analyse whether (non-)response to (ultra-)low dose of RTX at six months can be predicted at the moment of initiating retreatment.

## Methods

### Design

**Fig. 1:**
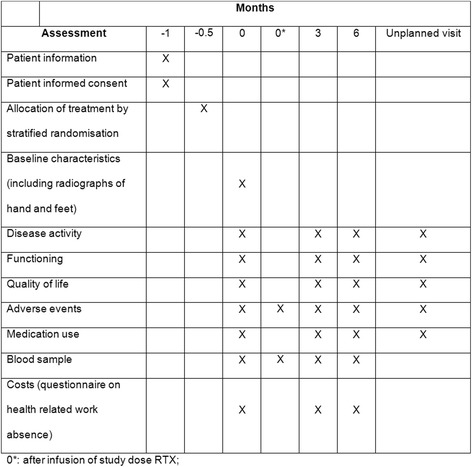
SPIRIT figure: trial visits and assessments

The REDO study (REtreatment with Rituximab in RhEmatoid arthritis: Disease Outcome after Dose Optimisation) is an investigator-driven, pragmatic, double-blind, non-inferiority RCT of six months’ duration (Fig. 1, SPIRIT checklist as Additional file 1). The trial is funded by two healthcare insurance companies in the Netherlands, Centraal Ziekenfonds (CZ) and Menzis, and independent from the manufacturer of RTX (Roche). The study is expected to be performed in at least three departments of rheumatology of hospitals in the Netherlands: the Sint Maartenskliniek, and Radboud University Medical Centre (Radboudumc) in Nijmegen; and Reade in Amsterdam. These centres together have approximately 400 RA patients being treated with RTX. Based on an earlier dose-tapering trial and similar inclusion criteria, we expect an inclusion percentage of 40%.

RA patients who are scheduled for RTX retreatment with standard low-dose RTX will be randomised into three groups: standard low dose (1 × 1000 mg) or one of the two ultra-low dose intervention groups (1 × 500 mg and 1 × 200 mg). Treatment response is assessed at three and six months (study end); thereafter, the allocation of patients will be revealed and treatment may be continued using any ultra-low or standard low dose of 1 × 1000 mg, at the discretion of the physician and patient in shared decision-making.

This report has been prepared in accordance to the SPIRIT guideline. The final report will follow the CONSORT criteria, including its extension to non-inferiority trials. The full study protocol is available as supplementary material. There are no publication restrictions and publication of the final study results will be performed in peer-reviewed journals as well as to lay press and patient organisations.

Important protocol changes will be communicated to the ethics committee and trial register. Privacy of patients will be protected according to Dutch law, WBP (‘wet bescherming persoonsgegevens’), by using anonymised data and restricting access to patient identification logs.

### Objectives

The primary objective of the REDO trial is to compare the difference in efficacy between two ultra-low doses (1 × 200 mg and 1 × 500 mg) and standard low dose (1 × 1000 mg) of RTX retreatment on the change in DAS28-CRP, compared to a pre-specified non-inferiority margin of 0.6 DAS28 points, at three and six months. Therefore, the study has four primary endpoints. Although we are aware that patients are sometimes treated with longer intervals than six months, showing non-inferiority at six months is relevant, for ultra-low RTX dose with at least six-month intervals is still a lower cumulative dose as standard low-dose 1000 mg every 9–12 months.

The main secondary objectives are: to assess the difference in efficacy between the two ultra-low dose interventions for the same outcomes; to compare the proportion of patients with a DAS28-CRP < 2.9 (low disease activity), DAS28-CRP < 2.4 (remission) and remission according to Boolean ACR/EULAR criteria at three-month and six-month follow-up; to assess the between-group differences in the change in functioning (HAQ-DI) and quality of life (EQ5D-5 L); and to compare proportion (cumulative incidence and incidence density) of patients developing (treatment-related) adverse events in each study group over the duration of the study, with special attention to infusion-related adverse events and infections. Furthermore, the cost-effectiveness of both ultra-low RTX doses and the conventional low dose are compared for the six-month study period. For prediction modelling, baseline factors (including RF/ACPA status, CD19+ B-cell count, serum RTX, serum anti-RTX) will be tested for associations with the outcome of DAS28-CRP low disease activity state at six months.

### Non-inferiority margin

In non-inferiority trials, the choice for a specific non-inferiority margin (NI margin) is critical for the interpretability of the study. This choice can be based on prior art (use of NI margin in comparable studies), expert opinion or data-driven, based on association with other (un)intended effects. We have found three non-inferiority studies that have used the DAS28 as a primary outcome measure. All three studies have chosen to use a NI margin of 0.6. [23–25]. Although no clear explanation is given by the authors regarding the rationale for this NI margin, a non-inferiority margin of 0.6 points in DAS28 seems a reasonable choice, as the error of measurement in DAS28 is 0.6 [26]. This error of measurement is used in the EULAR response criteria to denote the difference between a non-response and a moderate response in DAS28 [27]. Regarding assay sensitivity, the mean difference between placebo and RTX, added to MTX, in DAS28, is 1.2 according to a recent meta-analysis [1]. This means that the NI margin of 0.6 is sufficiently smaller than the treatment effect of RTX against placebo. We have therefore chosen to use this NI margin of 0.6, although it always remains debatable what an acceptable small NI margin is. This is especially important to prevent a situation where multiple non-inferiority studies are performed after each other, each using the non-inferior treatment from the last study as a comparator for a new treatment. In this context, although treatment B is non-inferior to A, and C is non-inferior to B, treatment C can in fact be inferior to A, the so called biocreep [28].

### Assay sensitivity

Since this is a non-inferiority trial, assay sensitivity – the ability to demonstrate inferiority with the chosen trial design – is an important issue. Assay sensitivity could be established by a placebo arm showing that not retreating with RTX is inferior to retreating with RTX. Considering it has been shown in earlier studies that the mean disease activity of patients will increase when not retreated with RTX [29], it seems unnecessary and unethical to include a placebo arm. Therefore, the comparator is a standard low-dose of RTX, while the group sizes should be large enough to gain a sufficient level of precision (see sample size calculation).

### Patients

Inclusion criteria for patients in this pragmatic study are as non-restrictive as possible. This is based on the underlying principle that the results of this trial should be generalisable to all RA patients who are doing well on their RTX treatment. We therefore include RA patients fulfilling either 2010 EULAR/ACR RA [30] and/or 1987 RA [31] criteria and/or having a clinical diagnosis of RA according to the treating rheumatologist, at any time point between start of the disease and inclusion.

Patients are eligible if they were treated at least once with regular low-dose RTX treatment in the last 18 months for RA, so in a dose of 1 × 1000 mg, 2 × 1000 mg or 2 × 500 mg, and had received no other bDMARDs after the last RTX dose. Patients treated with innovator RTX (MabThera®) as well as authorised RTX biosimilars in similar doses as conventional RTX will also be included.

It is somewhat difficult to operationalise the criterion that patients need to be doing well enough on RTX because of the variety of retreatment strategies that are used in clinical practice. We decided on at least six months of stable, low-disease activity after the last RTX infusion (operationalised by either DAS28-CRP < 2.9/DAS28-ESR < 3.2 or judgement of low-disease activity by a rheumatologist) and a current DAS28-CRP ≤ 3.5/DAS28-ESR ≤ 3.8. The latter criterion is added, because patients are often not retreated at fixed intervals, but are retreated either based on treat-to-target or on demand when disease activity increases. However, we do not want to generalise to patients being treated only when they flare severely, as it has been shown that the optimal strategy for RTX retreatment (although not completely clear yet) is either fixed interval or treat-to-target, but not treated only on demand. Also, a high SD in disease activity at study start would increase the required sample size.

Further inclusion criteria are chosen to ensure that we are able to study the participants and to measure the outcomes: patient informed consent; age ≥ 18 years and mentally competent; life expectancy > 6 months; no planned relocation out of reach of study centre; and able to read and communicate well in Dutch.

For generalisability reasons, exclusion criteria are kept minimal and only exclude patients with known (non-) response to ultra-low-dose RTX (below 1 × 1000 mg), to prevent selection bias, and current corticosteroid dosing above 10 mg per day prednisolone equivalent, because these patients should preferably first taper their corticosteroid.

### Patient recruitment

All eligible patients will be selected and approached based on information from the electronic health record according to the abovementioned inclusion and exclusion criteria. Patients will be asked to join this study by their treating rheumatologist using a letter accompanied with the patient information (including the informed consent form). Informed consent is obtained before patients receive the study medication and baseline data are collected.

### Randomisation and blinding

Participants will be allocated to the treatment groups at a ratio of 1:2:2 (1 × 1000 mg vs. 1 × 500 mg vs. 1 × 200 mg). The experimental groups are larger than the control group to increase experience with the lower dosing and with the additional benefit that a larger number of potential predictive factors for response can be studies in multivariate prediction modelling in the ultra-low-dose RTX groups.

Randomisation will be performed using a computerised randomisation procedure and stratified to ensure equal distributions of two possible effect modifiers for response to ultra-low-dose RTX, concomitant conventional DMARD use and RF/ACPA status. Patients will be randomised using block randomisation in variable block sizes (multiples of 5) to more closely achieve the intended allocation ratio and to ensure that the allocation of participants will not be predictable. Patients, physicians, nurses, researchers and data analyst/statistician will be blinded for treatment allocation. The allocation is kept in opaque, sequentially numbered envelopes; envelopes are sequentially assigned by the pharmacist to each next patient. The infusions for the study will be prepared by the hospital pharmacy based on the randomisation number, the physical appearance of the three interventions will be indiscriminate (see below). Unblinding is expected to be rarely necessary (all patients receive RTX and retreatment with 1000 mg is allowed when necessary), but is possible after consulting the coordinating centres pharmacist.

### Interventions

Patients allocated to the standard low-dose group will receive a (blinded) single 1000 mg RTX infusion according to the standard protocol for infusion of rituximab. Patients allocated to the ultra-low-dose groups will receive 500 mg or 200 mg. This dose will be diluted to the same volume as the standard low-dose infusion to ensure the blinding of the study, all premedication and procedures are identical to the standard low dose. Of note, the possible advantage of shorter infusion times cannot be assessed in our study, because this would lead to patients and healthcare providers being unblinded.

It is aimed to leave all other rheumatic treatment unaltered as much as possible during the study period. However, all treatment decisions are left to the discretion of the treating physician and (changes in) use of paracetamol (acetaminophen), tramadol, non-steroidal anti-inflammatory drugs (NSAIDs), oral corticosteroids and DMARDs are all allowed during this study to ensure good care. During each visit, patients are asked about the use of these medications. Suggested treatment in case of clear loss of response is escape treatment with an extra dose of 1 × 1000 mg RTX. This can be done without unblinding, since the authorised dose of RTX is 2 × 1000 mg per six months and no patients will exceed this dose as the maximum study dose is 1 × 1000 mg.

We have determined several medication changes that are defined as ‘treatment failure’. These changes are: receiving an extra dose of RTX within the six-month study period; receiving another bDMARD (thus switching to another type of bDMARD); and using corticosteroids in a dose > 10 mg/day. Starting a concomitant conventional synthetic (cs)DMARD during the study period is not considered a treatment failure. The reasoning behind this is that all included patients will have received these csDMARDs before, with little effect on their RA, and the concomitant csDMARD is generally given as an adjuvant to increase the effectiveness of RTX.

In case of treatment failure, the patients will remain in the study, but the last measure of disease activity and other outcomes will be used as outcome employing a ‘last observation carried forward’ strategy.

#### Assessments

At baseline, several characteristics of the patients will be measured, including demographics, disease and treatment characteristics. Also, possible predictors for response to ultra-low-dose RTX from peripheral blood will be collected, including (anti-)RTX drug levels and peripheral CD19 counts. Thereafter, visits will be performed at three and six months and when necessary in between (Fig. 1).

Several measures on disease activity will be collected during the study. The DAS28-CRP is a validated and widely accepted measure for RA disease activity and will be used as a primary outcome measure. It consists of four components: 28 tender joint count; 28 swollen joint count; CRP (mg/L); and patients VAS assessment of global disease activity (0–100) [26]. Remission is defined as DAS28-CRP < 2.4 and low disease activity by DAS28-CRP < 2.9 [32]. In addition, patient VAS assessment of pain, rheumatologist VAS assessment of global disease activity, acute phase reactants (CRP and ESR) and the OMERACT patient flare questionnaire are collected. To measure functioning of patients, the HAQ-DI, a validated instrument that is widely used in rheumatology is applied [33]. Quality of life is assessed using EQ5D-5 L, which is a validated instrument and comprises five questions and a visual analogue self-rating scale [34].

Adverse events are assessed at every visit during the study period and classified according to the Common Toxicity Criteria (CTC) [35]. In addition, we focus explicitly on infusion reactions and infectious events. Patients are asked to complete a short questionnaire after the RTX infusion on the occurrence of infusion-related adverse events. Medication use is charted using data from the electronic patient records on the use of DMARDs, corticosteroids and NSAIDs.

Costs will be calculated from a societal perspective. We will include the cost of outpatients’ clinic visits and telephone consultations, travel expenses for patients, costs of hospitalisation due to RA, costs due to health-related work absence and costs of medication during the six-month study period.

#### Sample size considerations and statistical analyses

The study has four primary endpoints; multiplicity over the primary endpoints will be protected by a fixed testing procedure. First, the non-inferiority of the 500 mg vs. 1000 mg at three months will be tested at *p* < 0.05 (two-sided). If this is statistically significant, then 500 mg vs. 1000 mg will be tested at *p* < 0.05 (two-sided) at six months. If that is statistically significant, then 200 mg vs. 1000 mg will be tested at *p* < 0.05 (two-sided) at three months and if that is statistically significant, the last test will be 200 mg vs. 1000 mg at *p* < 0.05 (two-sided) at six months. As we have four primary endpoints, we aim to have enough power for each at 95% for an NI margin of δ = 0.6. Under the worst-case scenario that these four are not correlated (the expectation is that they are positively correlated, see Table 1) and that the intervention is indeed non-inferior to the control condition, then the overall power for rejecting the null hypothesis of inferiority on all four is at least 95% × 95% × 95% × 95% = 81%. We calculated the sample size for one endpoint (e.g. the comparison of 500 vs. 1000 mg at six months). For 2:1 randomisation and a non-inferiority test assuming the true difference between treatments is 0, the total sample size for a t-test having a power 1-β when testing at significance level α (two-sided) and a non-inferiority margin δ is Ntot = (4.5)^2^ × (z_1-α/2_ + z_1-β_)^2^ × SD^2^/δ^2^, where z denotes the normal quantiles which are correct for non-small sample sizes. When correction for baseline is incorporated, this sample size is reduced by (1-r^2^) where r is the correlation in DAS28 between baseline and follow up (formula 7 with n = 1, π_0_ = 1/3, π_1_ = 2/3, and section 2.3 of Teerenstra S, et al. [36]). Note that the two groups then have sizes Ntot/3 and 2 × Ntot/3. To determine the correlation r between baseline and follow-up measurement of the DAS28, the following assumptions were used. Baseline DAS28 has a SD = 0.7 and the change from baseline to three (or six months) has a standard deviation of SDchange = 0.6 based on data from an earlier dose reduction trial [37]. As SD^2^change = 2 × (1-r) × SD^2^, it follows that r = 0.63. Then a total trial size of 80 participants would be enough. Table 1 illustrates the total trial size when the correlation between endpoints is smaller than anticipated.

**Table 1:** Total trial sample size at various correlations between endpoints

SD change	r	Sample size 1000 mg arm	Total trial size (5 x sample size in 1000 mg arm)
0.9	0.17	26	130
0.8	0.35	24	120
0.7	0.5	20	100
0.6	0.63	16	80

To protect for a too optimistic correlation, we therefore choose a total trial size of 130 and this is further increased to 140 patients to account for patient drop-out.

Primary analyses will be done per protocol (PP), as this is the most conservative approach for a non-inferiority study. In addition, analyses will be performed on an intention-to-treat basis (ITT). For PP analysis, we will include patients who have received the study medication and completed follow-up of six months or until treatment failure (and last observation of disease activity carried forward).

The primary endpoints will be tested using 95% confidence intervals based on linear regression with the change in DAS28-CRP as outcome, dose group as determinant and baseline values of DAS28-CRP as covariate (ANCOVA).

To find predictors (including age, sex, disease duration, RF/ACPA status, CD19+ B-cell count, serum RTX, serum anti-RTX), patients will be categorised into responders (DAS28-CRP < 2.9 at six months and no treatment failure) and non-responders (all other patients). The absolute number (and thus also proportion) of responders will determine the number of predictors that is admissible for analysis, according to the rule of ten-events-per-variable given that predictors are predetermined. Univariate logistic regression analysis will be performed for the admissible predictive factors, with a deliberately liberal *p* < 0.20 as selection criterion. Univariately significant variables are entered in a full multivariate logistic regression model, that is step-wise reduced until all *p* < 0.20. Internal validation and shrinkage will be performed using a bootstrapping procedure with 1000 repetitions. Performance of the multivariate predictive model will be evaluated using discrimination (area under the receiver operator curve) and calibration (calibration slope, calibration plot and Hosmer-Lemeshow test).

Costs will be calculated and quality-adjusted life-years (QALY) will be based on EuroQol-EQ5D-5 L utility scores. Decremental cost-effectiveness analyses (CEA) will be performed using bootstrap analyses; incremental net monetary benefit (iNMB) will be used to express cost-effectiveness at different willingness-to-pay (WTP) values in the range of €20,000–80,000 per QALY.

## Discussion

This study in summary is aimed at exploring the lower bound of effective RTX doses in RA, as there seems at least equipoise on whether ultra-low-dose RTX is effective in RA. The development of the current study protocol has some interesting aspects that should be discussed.

Because proper phase I/II dose-finding has not been done in RA for RTX in the development phase, and because RTX is already widely used in RA treatment, our study design shares some characteristics of both early dose-finding trials (small-/medium-sized blinded trial, medium follow-up, multiple dosing arms), as well as late pragmatic clinical studies (non-inferiority design, wide inclusion criteria, investigator driven, treat-to-target strategy, embedded in clinical practice, cost-effectiveness analyses). The lack of proper dose-finding may be caused by the fact that RTX was first developed for use in lymphoma. This means that the upper limit of toxicity was already known. Also, there was presumably less incentive for the pharmaceutical company to actively look for (much) lower effective RA dosing, as very different dosing schedules for between different diseases presents a problem when establishing drug prices. RTX was therefore eventually authorised in the same high dose for the treatment of RA. Indeed, due to the complex field of anti-cell or cytokine treatment – which is more pathophysiology than disease specific and might be very different in dosing across diseases – we expect this hybrid approach of post marketing investigator driven dose finding studies to be used more often in the near future.

Of note, our trial design precludes inference of the value of long-term repeated treatment strategies with ultra-low-dose RTX. For example, lower dosing might lead to shorter infusion intervals or ultra-low dose may not be effective enough after multiple retreatments. However, we believe that showing non-inferiority at six months would be a valuable step forward to further study an ultra-low-dose RTX retreatment strategy. Also, it will remain to be established whether inhibition of radiographic progression is not compromised using ultra-low-dose RTX.

In the specific case of ultra-low RTX dosing, some interesting developments might make the results of this study perhaps even more relevant. Recently, RTX – registered only after TNFi failure – has been shown to be similar in efficacy to TNFi in bDMARD-naïve patients [27]. Also, biosimilar RTX is expected to be available starting early 2017, at least in Europe. These two developments might make RTX as a first bDMARD a very realistic alternative. A promise of effective ultra-low-dose retreatment would further support this more prominent position of RTX in RA treatment.

### Trial status

The trial started on 15 December 2016 and is currently recruiting.

## Additional file

Additional file 1: SPIRIT checklist. (DOC 122 kb)

## Abbreviations

(b/cs) DMARD: (biological/conventional synthetic) Disease modifying anti rheumatic drug
ACPA: Anti-citrullinated peptide antibodies
ACR: American College of Rheumatology
CRP: C-reactive protein
CTC: Common toxicity criteria
DAS28: Disease Activity Score 28
ESR: Erythrocyte sedimentation rate
EULAR: European League Against Rheumatism
EUROQOL: European Quality of Life
HAQ DI: Health Assessment Questionnaire Disability Index
I/DCER: Incremental/decremental cost-effectiveness ratio
INMB: Incremental net monetary benefit
ITT: Intention to treat
MTX: Methotrexate
NSAID: Non-steroidal anti-inflammatory drug
PP: Per protocol
QALY: Quality-adjusted life-years
RA: Rheumatoid arthritis
RF: Rheumatoid factor
RTX: Rituximab
SD: Standard deviation
TNFi: Tumor necrosis factor inhibitor
WTP: Willingness to pay
